# Contributions of ABC Transporters and Cytochrome P450s to the Tolerance in *Antheraea pernyi* Against Beta-Cypermethrin

**DOI:** 10.3390/insects17040415

**Published:** 2026-04-13

**Authors:** Tianyi Zhang, Xin Chen, Junshan Chen, Peifeng Liu, Fengquan Liu, Miaomiao Chen, Liang Xu, Shiwen Zhao, Xisheng Li

**Affiliations:** Sericultural Research Institute of Liaoning Province, Dandong 118100, China; zhtiayi@163.com (T.Z.);

**Keywords:** ABC transporter, cytochrome P450, *Antheraea pernyi*, beta-cypermethrin, tolerance

## Abstract

*Antheraea pernyi* larvae growing in the wild suffer damage from the drift diffusion of insecticides used in surrounding farmland. In this study, we assessed the toxicity of beta-cypermethrin, chlorantraniliprole, imidacloprid, and thiamethoxam to different *A. pernyi* strains. It was found that the lowest LC_50_ value of the Liaocanda9 strain against beta-cypermethrin (0.008 mg/L) was significantly higher than that of the Kangda strain (0.0047 mg/L). Additionally, beta-cypermethrin exposure was associated with significant increases in detoxification and antioxidant enzyme activities in both strains. Transcriptomic analysis showed that differentially expressed genes (DEGs) were significantly enriched in pathways related to oxidoreductase activity and transmembrane transporter activity terms. Furthermore, these genes were differentially expressed following the beta-cypermethrin exposure. The dsRNA injection treatment effectively inhibited the expression of *P450* genes, thereby reducing the tolerance of *A. pernyi* against beta-cypermethrin by 25.93–55.56%. Molecular docking predicted that beta-cypermethrin bound to *ABCG1*, *ABCG5*, and *CYP9A22* with hydrogen bonds. These results indicate that ABC transporters and P450s contribute to the tolerance of *A. pernyi* against beta-cypermethrin.

## Simple Summary

In the wild, larvae of the moth *Antheraea pernyi* (a silkmoth species) are harmed by insecticides from nearby farms. This study evaluated the toxicity of four insecticides on different *Antheraea pernyi* strains, finding that the Liaocanda9 strain had a higher tolerance to beta-cypermethrin than the Kangda strain. Exposure to beta-cypermethrin increased detoxification and antioxidant enzyme activities in both strains. Transcriptomic analysis revealed that genes related to oxidoreductase and transmembrane transporter activities were differentially expressed after exposure. Injecting dsRNA reduced the expression of *P450* genes, decreasing the larvae’s tolerance to beta-cypermethrin by 25.93–55.56%. Molecular docking showed that beta-cypermethrin binds to ABCG1, ABCG5, and CYP9A22. These findings suggest that ABC transporters and P450s help *Antheraea pernyi* against beta-cypermethrin.

## 1. Introduction

Chinese tussah silkworm, *Antheraea pernyi* Guérin-Méneville (Lepidoptera: Saturniidae), is an economically significant insect that originated in China and is traditionally reared in the wild [1]. As a component of the “green gold” industry, its cocoons yield silk, while its pupae, rich in nutrients, find applications in the food and health supplement sectors, and the adult moths can be utilized for developing tonic medicines [2,3]. This has fostered a distinctive industrial system that integrates economic, ecological, and social benefits, serving as a crucial pillar for sustaining income growth among farmers in production regions [4]. The feeding behavior of *A. pernyi* exhibits marked specificity, with its larvae primarily relying on the leaves of oak trees (*Quercus mongolica*) as their core nutritional source [5]. However, severe pest infestations necessitate the use of insecticides in oak orchards, which inadvertently causes harm to the *A. pernyi* larvae that feed on these leaves [6]. Raising and selecting robust *A. pernyi* strains with tolerance to insecticides may help mitigate yield reductions [7]. Therefore, elucidating the molecular mechanisms underlying the insecticide tolerance of *A. pernyi* has become a focal point of current research in the breeding of *A. pernyi* strains.

The insect detoxification metabolic enzyme system serves as a crucial line of defense against insecticides [8]. Recent studies showed that silkworms develop tolerance to exogenous chemicals through the enzymes of cytochrome P450 monooxidase (P450), glutathione S-transferases (GST), carboxylesterases (CarE), and ATP-binding cassette transporters (ABC) [9]. It has been reported that the delayed response of P450 enzymes may be a potential cause for the increased susceptibility of silkworms to alpha-cypermethrin [10]. Research by Liu et al. [11] indicated that feeding silkworms an artificial diet resulted in induced P450 activity in their Malpighian tubules, suggesting an adaptation to dietary changes. It has been found that thiamethoxam treatment significantly increased P450 activity in silkworms within 24 h [12]. Findings reveal that after exposure to clothianidin, CarE and GST activities in silkworms showed an initial increase, followed by a decrease, and then a subsequent rise [7]. Guo et al. [13] demonstrated that avermectin induced the upregulation of ABC transporter expression in silkworms. Additionally, antioxidant enzymes such as superoxide dismutase (SOD), peroxidase (POD), and catalase (CAT) play central roles in pesticide tolerance, and adaptation in the *A. pernyi* [14]. Research by Shu et al. [15] revealed that under acidic intestinal conditions, silkworms activate the Duox-ROS defense system through increased antioxidant enzyme activity and reduced reactive oxygen species (ROS) levels. Studies have shown that treatment with the plant polyphenol resveratrol activated the SIRT7-FoxO-GST signaling pathway related to antioxidation in silkworms, thereby enhancing antioxidant enzyme activity to cope with adverse environmental conditions [16]. Although both *A. pernyi* and *Bombyx mori* belong to Lepidoptera, research on insecticide tolerance in the *A. pernyi* remains limited.

The detoxification mechanism of insects against xenobiotics involves the synthesis of detoxifying enzymes, signal transduction, and transcriptional regulation [17]. Understanding the molecular basis of insecticide tolerance mechanisms is crucial [9]. A comprehensive analysis of gene expression changes in insects following insecticide exposure facilitates the identification of key genes and regulatory networks associated with their defense against foreign substances [18]. Chen et al. [19] found that *ABCB1*, *ABCB3*, *ABCG11*, *ae43*, and *CYP6AE9* may be involved in the detoxification process of coumaphos in *A. pernyi*. Furthermore, *CYP4G25* in *A. pernyi* has been cloned, confirming its role in detoxification [20]. Mon et al. [21] constructed a gene co-expression network based on the *Bombyx mori* ovary-derived *BmN4* cell line and identified five genes whose knockdown led to abnormalities in chromosome dynamics and spindle morphology during mitosis. Research elucidated that the *Bombyx mori* lineage-specific gene *BmTrio* is positively correlated with the heterosis of silk yield traits [22]. The establishment of a gene expression model for silkworms induced by insecticides can precisely assess their tolerance to insecticides [23]. However, research on the molecular mechanisms of detoxification metabolism in *A. pernyi* remains limited, and the biological functions of related genes are not yet defined.

The hazards and drift diffusion of insecticides both cause loss of *A. pernyi* production, destroy its population structure, and affect its sustainable production [24]. It is very necessary to breed and screen the tolerance silkworm strains against pesticides. Here, the relatively tolerant Liaocanda9 strain and the susceptible Kangda strain were selected for toxicological bioassays of four commonly used insecticides. Based on the lowest LC_50_ value, the beta-cypermethrin was selected for further experimental analysis. The detoxification metabolic enzyme and antioxidant enzyme changes in Liaocanda9 and Kangda after beta-cypermethrin treatment were measured. Transcriptomics methods were used to screen related pathway genes, and the molecular processes in the bodies of silkworms to beta-cypermethrin treatment were further clarified by RT-qPCR, RNA interference and molecular docking. This study revealed the mechanism of silkworm tolerance against beta-cypermethrin, and provided a theoretical basis for the subsequent breeding of silkworm strain tolerance to pesticides by molecular means.

## 2. Materials and Methods

### 2.1. A. pernyi Collection

*A. pernyi* larvae used in this study comprised the robust Liaocanda9 strain and the Kangda strain, both of which had undergone multi-generation selective breeding. The silkworms were reared in oak groves at the Sericulture Science Research Institute of Liaoning province, China. Similarly sized silkworms on the second day of the third instar were selected. During collection, branches with silkworms were cut to avoid harming them. The collected silkworms were reared in an artificial climate laboratory at a temperature of 25 ± 2 °C and a humidity of 75 ± 5%.

### 2.2. Insecticides and Chemicals

We purchased 99% beta-cypermethrin {International Union of Pure and Applied Chemistry (IUPAC) name: (RS)-α-cyano-3-phenoxybenzyl (1R, 3R)-3-(2, 2-dichloroethenyl)-2, 2-dimethyl cyclopropanecarboxylate}; 97% chlorantraniliprole {IUPAC name: 3-Bromo-N-[4-chloro-2-methyl-6-[(methylamino)carbonyl]phenyl]-1-(3-chloro-2-pyridinyl)-1H-pyrazole-5-carboxamide}; 98% imidacloprid {IUPAC name: (E)-1-(6-chloro-3-pyridylmethyl)-N-nitroimidazolidin-2-ylideneamine}; and 98% thiamethoxam {IUPAC name: (EZ)-3-(2-Chloro-1, 3-thiazol-5-ylmethyl)-5-methyl-1, 3, 5-oxadiazinan-4-ylidene(nitro)amine} from Yuanye Bio-Technology Co., Ltd. (Shanghai, China).

Phosphate buffer solution (PBS), ethylene diamine tetraacetic acid (EDTA), dithiothreitol (DTT), phenyl methyl sulfonyl fluoride (PMSF), reduced glutathione (GSH) glycerol, α-naphthyl acetate (α-NA), 7-ethoxycoumarin (ECOD), and nicotinamide adenine dinucleotide phosphate (NADPH) were purchased from Solarbio Technology Co., Ltd. (Beijing, China).

### 2.3. Toxicological Bioassays

To determine the toxicity of different insecticides to the Liaocanda9 and Kangda strains of silkworms, a leaf-dipping bioassay method was employed [7]. The insecticides were weighed and dissolved in acetone to prepare a stock solution with a concentration of 1000 mg/L for standby use. Subsequently, the stock solution was diluted to create working solutions with five gradient concentrations (beta-cypermethrin: 0.003, 0.006, 0.009, 0.012, 0.015 mg/L; chlorantraniliprole: 5, 10, 15, 20, 25 mg/L; imidacloprid: 0.2, 0.4, 0.6, 0.8, 1 mg/L; thiamethoxam: 10, 20, 30, 40, 50 mg/L). Fresh oak leaves were then collected and immersed in the working solutions for 20 s before being removed and air-dried.

For each treatment, 20 healthy 3rd-instar silkworms of similar size, on the second day of the instar, were used for the bioassay. Before the bioassay, silkworms were subjected to a 4 h fasting period. After that, they were fed with oak leaves containing the insecticides. Oak leaves with an equivalent amount of acetone served as the control treatment. Each experimental group included three biological replicates, and the mortality rate of the silkworms was recorded 48 h after treatment.

### 2.4. Enzymatic Assays

To investigate the impacts of beta-cypermethrin on the activities of detoxification and metabolic enzymes (P450, CarE, and GST) and antioxidant enzymes (SOD, POD, and CAT) in Liaocanda9 and Kangda strains, the following experimental procedure was carried out. After feeding the silkworms with LC_50_ beta-cypermethrin (Liaocanda9: 0.008 mg/L; Kangda: 0.0047 mg/L) for 48 h, the surviving silkworms were collected and stored at −80 °C. Single silkworm was placed in a mortar with 10 mL of precooled phosphate buffer and grinded on ice. The supernatant was collected after centrifugation as the crude enzyme solution, which was stored at −80 °C. Each experiment was repeated three times.

P450 activity was measured following the method described by Rosenberg [25] with some modifications. Total reaction volume was 140 µL, comprising 80 µL of 0.5 mM ECOD and 50 µL of crude enzyme extract. The mixture was incubated with shaking at 30 °C for 30 min, followed by the addition of 10 µL of 9.6 mM NADPH. Then absorbance was measured using a multifunctional microplate reader with an excitation wavelength of 380 nm and an emission wavelength of 460 nm at 30 °C, recording data every 60 s for 15 min. GST activity was determined according to the method of Enayati [26] with some adaptations. Total reaction volume was 220 µL, consisting of 20 µL of crude enzyme extract, 180 µL of CDNB (15 mmol/L), and 20 µL of reduced glutathione GSH (30 mmol/L). The absorbance changes were recorded with a wavelength of 340 nm at 25 °C, recording data every 60 s for 20 min. CarE activity was assayed following the method of Li [27] with some modifications. Total reaction system was 210 µL, including 150 µL of substrate (0.3 mM α-naphthol; 3 µM physostigmine), 18 µL of PBS, and 2 µL of crude enzyme extract. After incubation at 30 °C for 15 min, 30 µL of a benzoylaminodimethoxyanilinediaz (0.4 mM) was added. The absorbance changes were recorded with a wavelength of 600 nm at 27 °C, recording data every 60 s for 10 min. The activities of SOD, POD, and CAT were determined according to the instructions provided in the corresponding assay kits purchased from Solarbio Technology Co., Ltd. (Beijing, China). Specifically, SOD activity was measured using the nitroblue tetrazolium method [28], POD activity was measured using the guaiacol method [29], and CAT activity was measured as decomposition of H_2_O_2_, monitored by decreasing absorbance at 240 nm [30].

### 2.5. RNA Sequencing

The transcriptomic changes in Liaocanda9 and Kangda strains under the induction of LC_30_ beta-cypermethrin were determined. Four experimental groups were included: Liaocanda9 control (0 mg/L), Liaocanda9 treatment (0.0052 mg/L), Kangda control (0 mg/L), and Kangda treatment (0.0031 mg/L). Each treatment after 48 h consisted of one third instar silkworm and was repeated 3 times; the individuals were carefully selected to be genetically homogeneous and raised under strictly controlled conditions. Total RNA was extracted from the tissue samples using QIAzol Lysis Reagent (Qiagen, Hilden, Germany). Libraries were generated using the Illumina^®^ Stranded mRNA Prep, Ligation (Illumina, San Diego, CA, USA), and were sequenced on the DNBSEQ-T7 platform. The quality of the raw sequencing data was controlled using fastp software version 0.19.5 (https://github.com/OpenGene/fastp, accessed on 4 June 2025) [31]. Reads were aligned to the reference genome GWHABGR00000000 (https://ngdc.cncb.ac.cn/gwh, accessed on 4 June 2025) [32] using HiSat2 software version 2.1.0 (http://ccb.jhu.edu/software/hisat2/index.shtml, accessed on 4 June 2025). The expression levels of samples were analyzed using RSEM (http://deweylab.github.io/RSEM/, accessed on 4 June 2025) [33]. Differentially expressed genes (DEGs) were screened based on the criteria of FDR < 0.05 |log_2_FC| ≥ 1 using DESeq2 software version 1.12.3 (http://bioconductor.org/packages/stats/bioc/DESeq2/, accessed on 4 June 2025) [34]. Enrichment analysis of DEGs was performed using GO (http://geneontology.org/, accessed on 4 June 2025) [35] and KEGG (https://www.genome.jp/kegg/, accessed on 4 June 2025) [36].

### 2.6. RT-qPCR Validation

The third instar larvae of Liaocanda9 and Kangda strains were treated with LC_50_ beta-cypermethrin (Liaocanda9: 0.008 mg/L; Kangda: 0.0047 mg/L) for 48 h and then stored at −80 °C. Based on the transcriptome data, the related DEGs were screened, and the primers were designed by Primer3 (https://primer3.ut.ee/, accessed on 4 June 2025) (Table S1). Total RNA was used for cDNA synthesis with the TransScript First-Strand cDNA Synthesis kit (AiDLAB Biotech, Beijing, China). RT-qPCR was then performed using the SYBR Green QPCR Mix (Sichuan KeJin, Deyang, China). Based on the results of our pre-experiment, *18S rRNA* was selected as the internal reference gene, and the relative expression levels of the genes were calculated by the 2^−△△Ct^ method [37].

### 2.7. RNA Interference

Gene sequences were obtained based on the transcriptome to design primers with T7 promoters (Table S2). The dsRNA was synthesized using T7 RNAi transcription kit (Vazyme Biotech, Nanjing, China) following the manufacturer’s instructions. The concentration of dsRNA was detected by using a Micro Drop ultramicro spectrophotometer (Mapada, Shanghai, China) and dissolved to 1000 ng/μL in ddH_2_O [38]. Sixty healthy 3rd-instar silkworms of a similar size were selected and randomly divided into 3 groups, each group of 20 silkworms. A total of 10 μL of dsRNA was absorbed by a microsyringe, then paralleled into the membrane between the first and third thoracic segments of the larvae’s back, and slowly injected subcutaneously. The needle should not be inserted too deeply to avoid puncturing the larvae. Injection of the same amount of dsEGFP and ddH_2_O was used as control. The silkworms were placed on oak leaves to recover. Then, silkworms were collected at 24 h, 48 h, and 72 h after injection of dsRNA, and used for RT-qPCR detection of gene expression after being frozen by liquid nitrogen; each treatment was repeated three times. Silkworms were starved for 4 h after injection of dsRNA and then fed with the oak leaves containing LC_50_ beta-cypermethrin (0.008 mg/L) for bioassay. Mortality was recorded at 48 h after treatment. Each treatment used 20 silkworms and was repeated three times.

### 2.8. Molecular Docking of A. pernyi Proteins

To investigate the interaction between beta-cypermethrin and *A. pernyi* proteins, the simulated molecular docking experiment was conducted. All input files were prepared using the Autodock Vina software version 4.2 [39]. The sdf file of beta-cypermethrin was obtained from the PubChem database. Subsequently, energy minimization, hydrogen addition, and charge correction were performed on this file. The structure of the *A. pernyi* protein was preprocessed, which involved removing water molecules from the protein crystal structure, adding missing hydrogen atoms to protein residues, and optimizing the protein conformation. Molecular docking was then carried out, with beta-cypermethrin serving as the ligand and the *A. pernyi* protein as the receptor. Full-atom docking approach was selected to evaluate their binding modes. The binding affinities were classified into three levels: extremely weak or no binding (affinity > −4 kcal/mol), moderate binding (−7 kcal/mol < affinity ≤ −4 kcal/mol), and strong binding (affinity ≤ −7 kcal/mol). Molecular docking results were visualized using the Pymol software version 3.1 [40].

### 2.9. Data Analysis

The median lethal concentration (LC_50_) and corresponding 95% confidence limit (95% CL) were calculated using the PoloPlus software version 1.0 [41]. The *p*-values were analyzed with the GraphPad InStat 3.0 software. Statistical analysis of the mean values and standard errors of the data was conducted using SPSS 22.0 software. One-way analysis of variance (ANOVA) was performed at a significance level of *p* < 0.05, followed by Tukey’s post hoc test.

## 3. Results

### 3.1. Bioassays of A. pernyi

**Figure 1:**
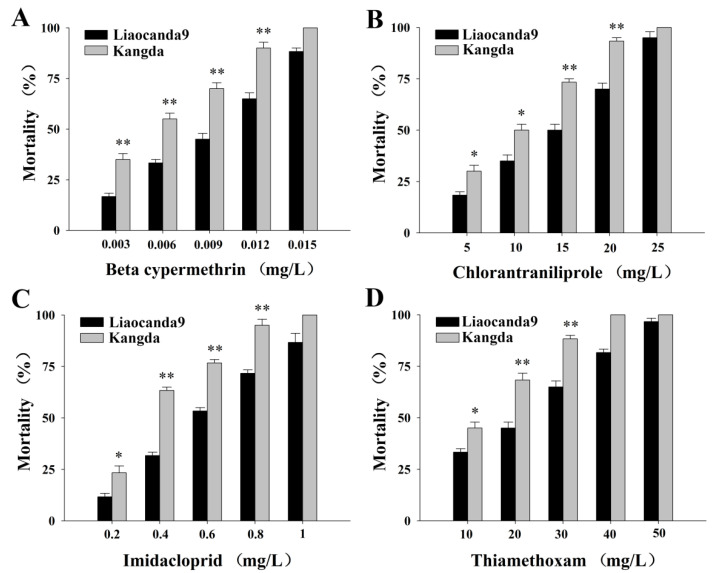
The mortality of *A. pernyi* larvae to four insecticides. (**A**) Beta-cypermethrin. (**B**) Chloraniliprole. (**C**) Imidacloprid. (**D**) Thiamethoxam. The sample size for each group is 60. The significance thresholds are set as follows: * indicates *p* < 0.05 and ** indicates *p* < 0.01. Comparisons are made between Liaocanda9 and Kangda strain. Error bars represent the standard error of the mean (SEM).

To clarify the sensitivity of *A. pernyi* larvae to insecticides, the toxicity of four insecticides against the Liaocanda9 and Kangda strains was determined. The results in Table 1 showed that the LC_50_ values of beta-cypermethrin for the Liaocanda9 and Kangda strains were 0.008 mg/L and 0.0047 mg/L, respectively, indicating higher toxicity compared to the other three insecticides. The LC_50_ values of insecticides for the Liaocanda9 strain were higher than those for the Kangda strain with not-overlapping 95% confidence limits. In addition, the mortality of *A. pernyi* larvae from insecticides exhibited a significant dose-dependent pattern (Figure 1). When treated with the same concentration of insecticides, the mortality rate of the Liaocanda9 strain was significantly lower than that of the Kangda strain. These results suggest that the Liaocanda9 strain exhibits higher tolerance against four insecticides.

### 3.2. Enzyme Activity Determination

The results in Table 2 show that after treatment with beta-cypermethrin, the activities of P450, GST, and CarE in both Liaocanda9 and Kangda strains significantly increased (*p* < 0.05). Moreover, the activities of P450, GST, and CarE in the Liaocanda9 strain were notably higher than those in the Kangda strain (*p* < 0.05). P450, GST, and CarE enzymes may play important roles in the detoxification and metabolism of *A. pernyi* against beta-cypermethrin.

The results in Table 3 also show that following beta-cypermethrin treatment, the activities of SOD, POD, and CAT in both Liaocanda9 and Kangda strains significantly increased (*p* < 0.05). Additionally, the activities of SOD, POD, and CAT in the Liaocanda9 strain were significantly greater than those in the Kangda strain (*p* < 0.05). These findings suggest that the antioxidant enzymes SOD, POD, and CAT may contribute to the tolerance of *A. pernyi* to beta-cypermethrin.

### 3.3. Transcriptome Analysis

The results of transcriptome sequencing of 12 samples showed that a total of 79 b of clean data was obtained, with each sample having clean data of more than 6.21 Gb; the percentage of Q20 bases was above 99.24%, the percentage of Q30 bases was above 96.07%, and the GC content was 45.15–48.46%. The sequence alignment mapping rate was above 87.98% (Table S3).

**Figure 2:**
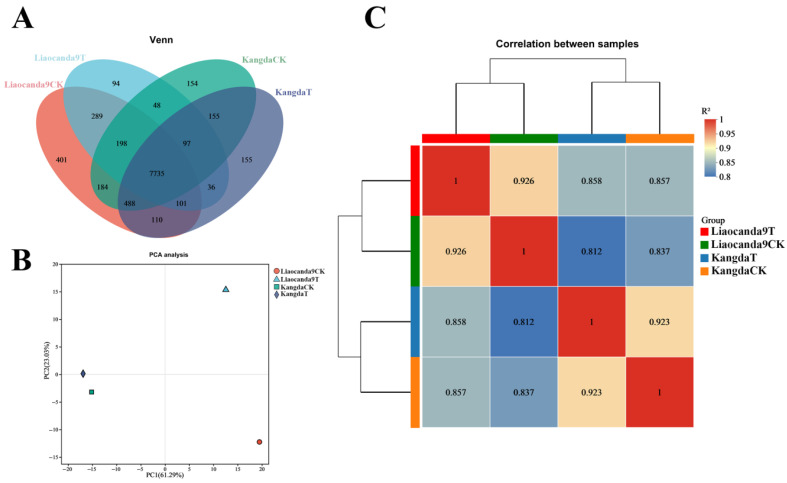
Transcriptome analysis of beta-cypermethrin-induced Liaocanda9 and Kangda strains. (**A**) Venn analysis. (**B**) Principal component analysis chart. (**C**) Correlation clustering heat map between different samples.

Venn analysis was conducted to illustrate the distribution of gene numbers across different sample groups (Figure 2A). It was found that 7.65% of the differentially expressed genes were unique to Liaocanda9, 4.52% were unique to Kangda, and 87.83% were shared between Liaocanda9 and Kangda. Additionally, beta-cypermethrin may induce differential expression in 2.69% and 3.92% of genes in Liaocanda9 and Kangda, respectively.

Principal component analysis (PCA) was performed on the gene expression levels of each sample (Figure 2B). The results showed that the data variability accounting for PC1 principal component factor was the largest, at 61.29%, indicating the differences in gene expression levels between Liaocanda9 and Kangda groups. The data variability accounting for PC2 principal component factor was 23.03%, highlighting differences in gene expression levels between the beta-cypermethrin-treated group and the control group.

Pairwise correlation analysis of gene expression levels across samples revealed that the correlation coefficients between the treated and control groups in Liaocanda9 and Kangda were 0.926 and 0.923, respectively, indicating that beta-cypermethrin treatment had a certain impact on gene expression levels in both Liaocanda9 and Kangda (Figure 2C). The correlation coefficient between the Liaocanda9 and Kangda groups was 0.837, reflecting differences in expression levels between the strains.

**Figure 3:**
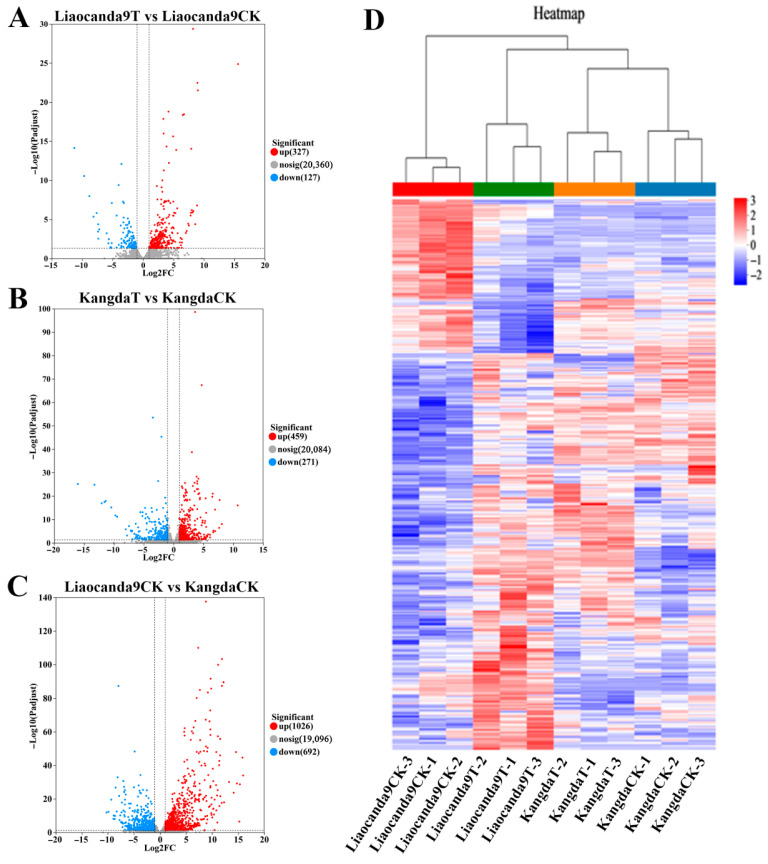
Analysis of beta-cypermethrin-induced Liaocanda9 and Kangda strain DEGs. (**A**–**C**) Volcano map of DEG distribution, with standard of FDR < 0.05, |log_2_FC| > 1 (dotted line); red: upregulation; blue: downregulation; gray: not significant. (**D**) Clustering heat map of DEGs; red: positive correlation; blue: negative correlation.

With a standard of *p* < 0.05, |log_2_FC| > 1, DEGs were screened across groups. Compared to the control group, 454 DEGs were identified in the beta-cypermethrin-treated Liaocanda9 group, with 327 genes upregulated and 127 genes downregulated (Figure 3A). In the beta-cypermethrin-treated Kangda group, 730 DEGs were identified, with 459 genes upregulated and 271 genes downregulated (Figure 3B). As shown in Figure 3C, there were 1718 DEGs identified, including 1026 upregulated genes and 692 downregulated genes between the Liaocanda9 and Kangda groups. Based on the DEGs, the three replicate samples in each group exhibited clustering relationships (Figure 3D).

### 3.4. GO and KEGG Enrichment Analysis of DEGs

**Figure 4:**
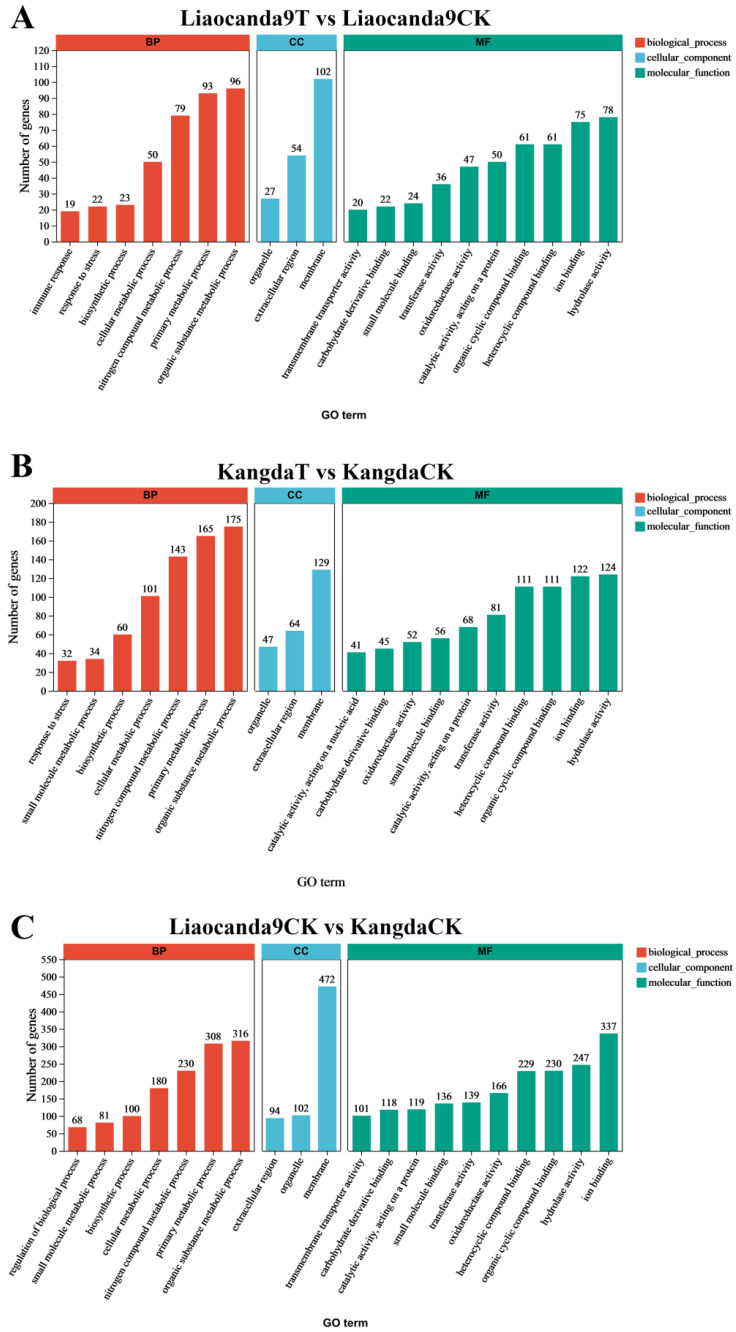
GO function annotation of DEGs. (**A**) Liaocanda9T vs. Liaocanda9CK. (**B**) KangdaT vs. KangdaCK. (**C**) Liaocanda9CK vs. KangdaCK. Red: biological process; blue: cellular component; green: molecular function.

Enrichment analysis of DEGs was conducted using Gene Ontology (GO) terms (Figure 4). When comparing the beta-cypermethrin-treated group and the control group of Liaocanda9, the DEGs were primarily enriched in response to stress (GO: 0006950), immune response (GO: 0006955), oxidoreductase activity (GO: 0016491), and transmembrane transporter activity (GO: 0022857) (Figure 4A). In contrast, when comparing the beta-cypermethrin-treated group and the control group of Kangda, the DEGs were mainly enriched in response to stress and oxidoreductase activity (Figure 4B). Moreover, compared to Kangda, the DEGs in Liaocanda9 exhibited greater enrichment in oxidoreductase activity and transmembrane transporter activity (Figure 4C).

**Figure 5:**
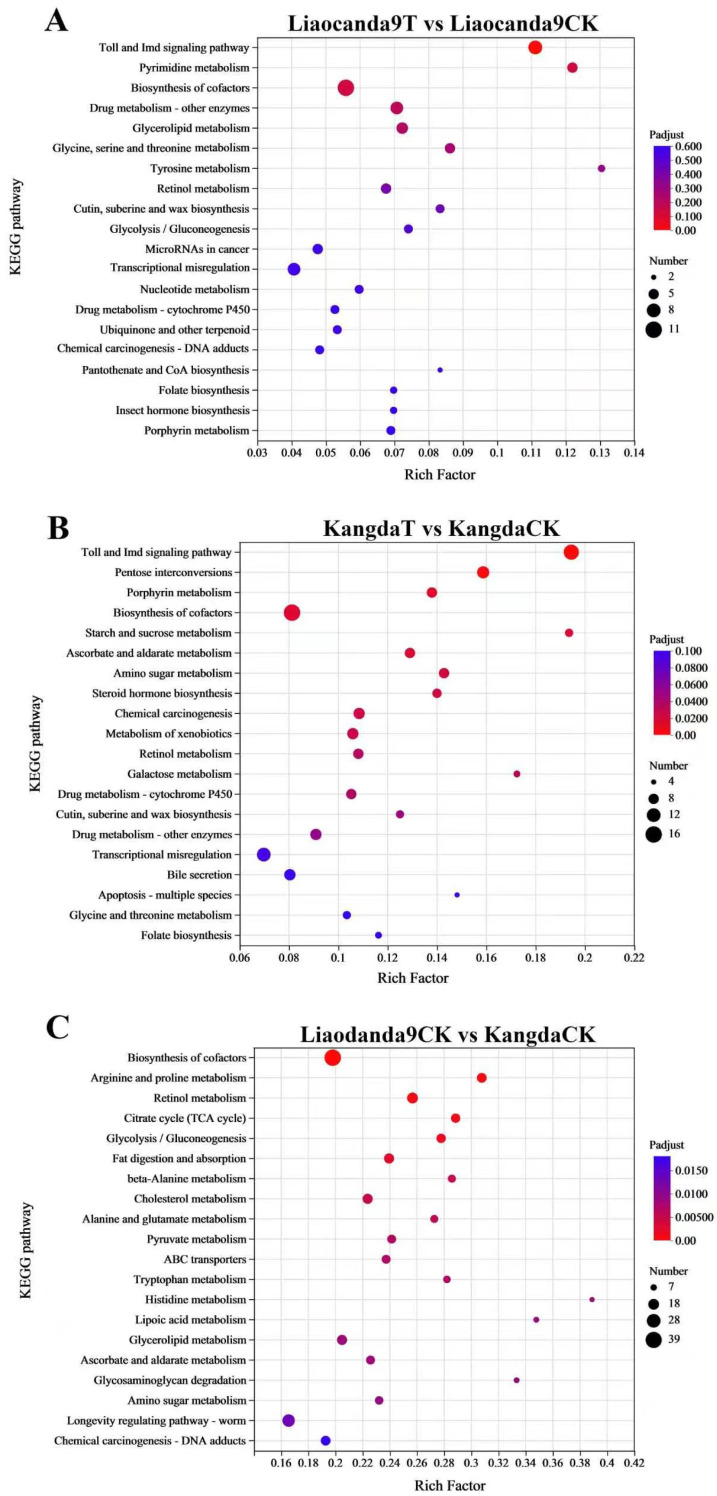
KEGG pathway analysis of DEGs. (**A**) Liaocanda9T vs. Liaocanda9CK. (**B**) KangdaT vs. KangdaCK. (**C**) Liaocanda9CK vs. KangdaCK. The significance of pathways is indicated by the q-value (color bar), the rich factor (X-axis), and the circles indicating the numbers of DEGs.

The KEGG database was employed to classify the DEGs based on their functional pathways or roles (Figure 5). Compared to the control group, in the beta-cypermethrin-treated group of Liaocanda9, DEGs were primarily enriched in the pathways of drug metabolism–other enzymes (map00983), drug metabolism–cytochrome P450 (map00982), and chemical carcinogenesis–DNA adducts (map05204) (Figure 5A). In comparison to the control group, in the beta-cypermethrin-treated group of Kangda, the DEGs were mainly enriched in the pathways of chemical carcinogenesis–DNA adducts, metabolism of xenobiotics by cytochrome P450 (map00980), drug metabolism–cytochrome P450, and drug metabolism–other enzymes (Figure 5B). Additionally, compared to the group of Kangda, the DEGs in the group of Liaocanda9 showed greater enrichment in the ABC transporters (map02010) and chemical carcinogenesis–DNA adducts pathways (Figure 5C).

### 3.5. RT-qPCR Validation of DEGs Related to Tolerance

**Figure 6:**
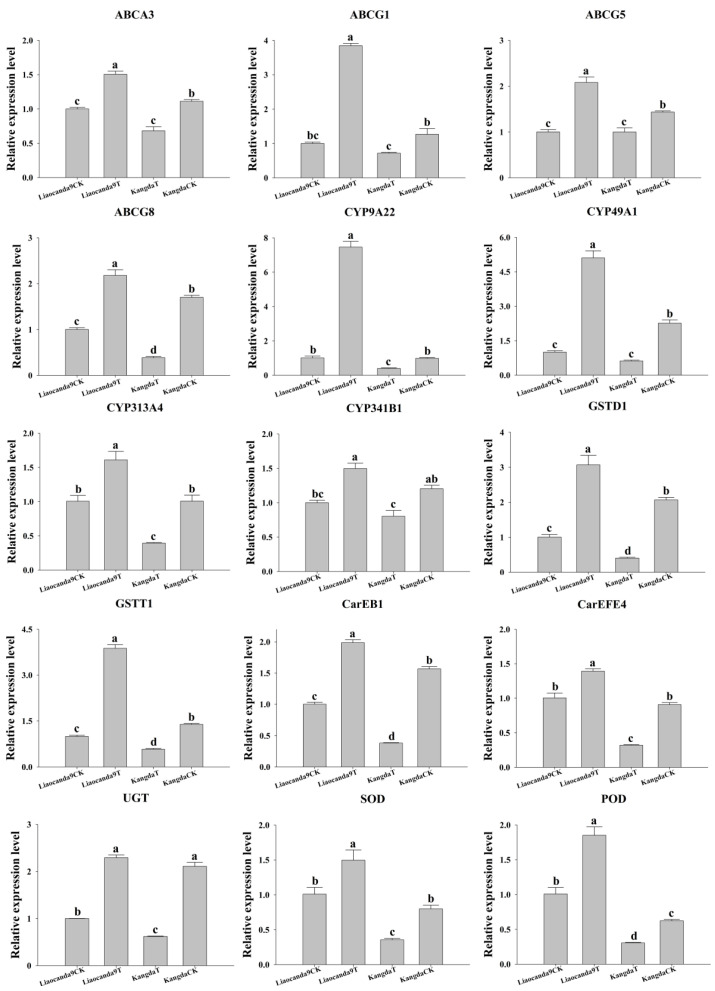
RT-qPCR validation of DEGs related to detoxification metabolism in Liaocanda9 and Kangda strains. Data were normalized by using *18S rRNA*. Different letters above bars indicate significant differences based on ANOVA followed by Tukey’s HSD post hoc test (*p* < 0.05).

To elucidate the molecular mechanisms underlying the differential tolerance of *A. pernyi* against beta-cypermethrin, RT-qPCR was employed to validate DEGs identified from transcriptome sequencing data. These genes included ABC transporters, cytochrome P450s, glutathione S-transferases, carboxylesterases, UDP-glucosyltransferases, superoxide dismutases, and peroxidases (Figure 6). Compared to the control group, the relative expression levels of DEGs in Liaocanda9 and Kangda strains after beta-cypermethrin treatment were upregulated by 1.39–7.38-fold and 1.43–5.08-fold, respectively (*p* < 0.05). The upregulation of these genes is consistent with a potential contribution to the higher tolerance of the Liaocanda9 strain against beta-cypermethrin.

### 3.6. RNA Interference of ABC and P450 Genes

**Figure 7:**
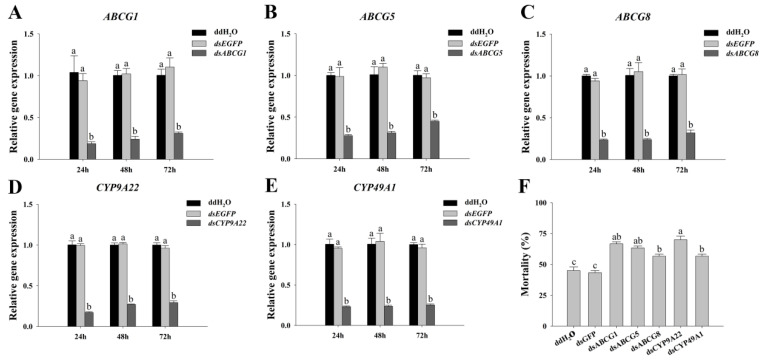
Interference efficiency of *ABCG1* (**A**), *ABCG5* (**B**), *ABCG8* (**C**), *CYP9A22* (**D**), and *CYP49A1* (**E**) by RNAi. Mortality at 24 h of dsRNA injection in Liaocanda9 strain after treatment with LC_50_ beta-cypermethrin (**F**). Different letters above bars indicate significant differences based on ANOVA followed by Tukey’s HSD post hoc test (*p* < 0.05).

To investigate the function of ABC transporter and cytochrome *P450* genes, dsRNA was synthesized and injected into the Liaocanda9 strain for RNA interference (RNAi). The RNAi efficiency results revealed that, after dsRNA injection for 24 h, the relative expression levels of *ABCG1*, *ABCG5*, *ABCG8*, *CYP9A22*, and *CYP49A1* were significantly reduced by 80.12%, 71.91%, 75.11%, 82.73%, and 75.74% (*p* < 0.05), respectively, compared to the control group (Figure 7A–E). Even after dsRNA injection for 72 h, the expression of these genes remained significantly reduced, with relative expression levels of *ABCG1*, *ABCG5*, *ABCG8*, *CYP9A22*, and *CYP49A1* decreasing by 71.76%, 53.67%, 68.70%, 69.57%, and 73.65% (*p* < 0.05), respectively.

Furthermore, the mortality rate of *A. pernyi* in response to beta-cypermethrin was assessed at 24 h after dsRNA injection. The results indicated that, compared to the control group, the mortality rates of silkworms treated with *dsABCG1*, *dsABCG5*, *dsABCG8*, *dsCYP9A22*, and *dsCYP49A1* significantly increased by 48.15%, 40.74%, 25.93%, 55.56%, and 25.93% (*p* < 0.05), respectively. Interference with these five genes significantly reduced the tolerance of *A. pernyi* against beta-cypermethrin. These findings suggest that *ABCG1*, *ABCG5*, *ABCG8*, *CYP9A22*, and *CYP49A1* play important roles in the defense mechanism of *A. pernyi* against beta-cypermethrin.

### 3.7. Molecular Docking

**Figure 8:**
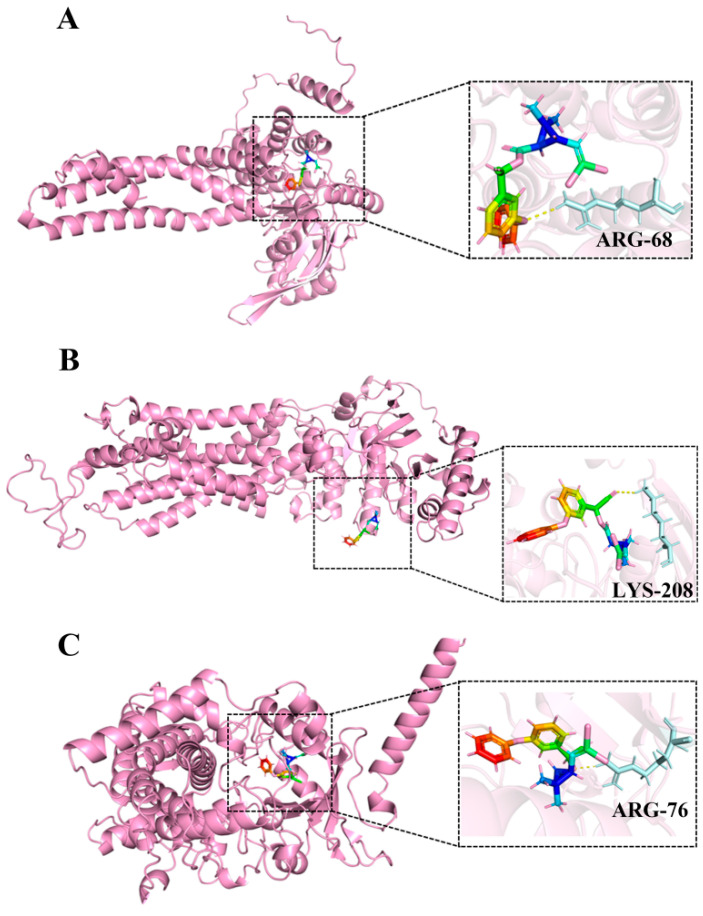
Molecular docking between beta-cypermethrin and *A. pernyi* protein in silico predictions without experimental validation. (**A**) ABCG1, (**B**) ABCG5, (**C**) CYP9A22.

Molecular docking models were constructed to predict the interactions between beta-cypermethrin and *A. pernyi* proteins in silico predictions without experimental validation. The molecular docking scores for beta-cypermethrin with ABCG1, ABCG5, and CYP9A22 were −9.4 kcal/mol, −8.4 kcal/mol, and −9.1 kcal/mol, respectively, indicating strong binding capabilities between beta-cypermethrin and proteins. Figure 8A illustrates that beta-cypermethrin interacts with the amino acid residue *Arg68* of ABCG1 via hydrogen bonds. Figure 8B demonstrates that beta-cypermethrin forms hydrogen bonds with the amino acid residue *Lys208* of ABCG5. Figure 8C reveals that beta-cypermethrin interacts with the amino acid residue *Arg76* of the CYP9A22 protein through hydrogen bonds. These results provide insights into the predicted binding modes of beta-cypermethrin with *A. pernyi* proteins.

## 4. Discussion

The extensive use of insecticides in agricultural production presents new challenges for the rearing of *A. pernyi*. Pesticide drift and residues cause phytotoxicity to *A. pernyi* larvae, severely impacting cocoon yield and quality [42]. Previous reports indicate that pesticide contamination from adjacent farmland can indirectly harm sericulture [43]. In this study, we evaluated the toxicity of four commonly used insecticides to *A. pernyi*, revealing that the larvae exhibit high sensitivity to all tested compounds. Notably, beta-cypermethrin showed the lowest LC_50_ value, indicating a high risk to *A. pernyi*. Even extremely low concentrations of insecticide exposure can be lethal to silkworms, suggesting that trace residues, nearly undetectable in the environment, are sufficient to devastate *A. pernyi* strains [7]. One report assessed the effects of chlorfenapyr on larval weight, cocoon weight, and silk yield in silkworms, demonstrating its threat to insect development and silk production [44].

Changes in enzymatic activity constitute a primary defense line for insects against xenobiotics and serve as a key biochemical basis for the development of insecticide tolerance [45]. It has been reported that the fat body of *A. pernyi* can enhance the activity of redox system enzymes by promoting the expression of SOD, CAT, GST, and glutathione peroxidase (GSH-Px), thereby improving the response of the organism to uranium-induced stress [46]. A correlation exists between insecticide tolerance in insects and the activity of their detoxification and antioxidant enzymes [47]. Qadri et al. [48] found that silkworms utilize endogenous antioxidants to scavenge reactive oxygen species generated by dimethoate treatment. Here, we observed that beta-cypermethrin treatment activated responses in both detoxification and antioxidant enzyme activities in *A. pernyi*. Furthermore, enzyme activities in different *A. pernyi* strains showed a positive correlation with their insecticide tolerance.

The evolution of insecticide tolerance in insects is influenced by the complex remodeling of gene expression and regulatory networks [45]. Insecticides act as potent selective pressures, driving genomic changes that involve adaptive adjustments ranging from the differential expression of detoxification enzyme-coding genes to signal transduction and transcriptional regulation networks [49]. Research on the model organism *Bombyx mori* has provided mature methodologies and profound insights [19]. Wang et al. [50] discovered that *B. mori* employs a midgut immune regulatory mechanism involving immune deficiency, antimicrobial peptides, and the JAK/STAT pathway to counteract broflanilide toxicity. Although genetic research on tolerance exists for *A. pernyi*, its molecular mechanisms remain unclear [51]. In this study, DEGs following beta-cypermethrin treatment were primarily enriched in pathways such as ABC transporters, drug metabolism–cytochrome P450, and chemical carcinogenesis–DNA adducts in *A. pernyi*. Additionally, DEGs were more significantly enriched in Gene Ontology terms related to oxidoreductase activity and transmembrane transporter activity in the more tolerant Liaocanda9 strain. These DEGs may represent candidate factors that contribute to insecticide tolerance in *A. pernyi*.

During the prolonged struggle against synthetic insecticides, insects have evolved a sophisticated, efficient, and highly coordinated molecular defense system [52]. Insect genomes often contain dozens or even hundreds of P450 genes, divided into multiple families and subfamilies [53]. This diversity enables insects to respond to various chemical challenges by expressing different combinations of P450 enzymes [54]. Enhanced P450s is a core mechanism inducing insect tolerance; upon perceiving insecticide stress, insects rapidly upregulate the transcription of relevant *P450* genes through specific signaling pathways [55]. Studies have shown that O-Vanillin binds to the *B. mori* cytochrome P450 subfamily protein *CYP9A19* to alleviate the toxicity of dinotefuran [56]. Li et al. [57] found that *CYP6B5*, *CYP9f2*, and *CYP6B6* are involved in the detoxification metabolism of chlorfenapyr in *B. mori*. Furthermore, ABC transporters actively efflux polar products from Phase I metabolism or even partially unmodified parent toxins out of cells, effectively reducing their intracellular accumulation and preventing them from reaching toxic thresholds [58]. These transporters work in close conjunction with metabolic enzymes, as P450 metabolites are often preferred substrates for ABC transporters [59]. Research indicates that genes in the *B. mori* ABC-B, -C, and -G subfamilies encode homologs of P-glycoprotein, multidrug resistance protein, and breast cancer resistance protein [60]. Feng et al. [61] demonstrated that the *B. mori* ABC transporter *BmWh2* inhibits nucleopolyhedrovirus proliferation by enhancing antithymocyte globulin expression. Studies have shown that avermectin treatment upregulated the expression of ABC transporters and detoxification metabolic genes in *B. mori* [13]. In this study, in vivo injection of dsRNA targeting *ABCG1*, *ABCG5*, *ABCG8*, *CYP9A22*, and *CYP49A1* significantly increased the mortality of *A. pernyi* exposed to beta-cypermethrin, further confirming the crucial roles these five genes play in tolerance. Additionally, molecular docking results support direct binding between *A. pernyi* proteins and the beta-cypermethrin molecule. The strong binding of beta-cypermethrin to ABCG1, ABCG5, and CYP9A22 likely disrupts their normal functions in detoxification, transport, or metabolism, leading to an increased susceptibility of *A. pernyi* to the insecticide.

## 5. Conclusions

The development of insecticide tolerance in *A. pernyi* mitigates, to some extent, the impact of toxicity on larvae during wild rearing. Both detoxification metabolic enzymes and antioxidant enzymes are involved in the defense against beta-cypermethrin. Comparative transcriptome analysis of beta-cypermethrin-treated *A. pernyi* identified changes in related metabolic pathways, with the response of ABC transporter and P450 genes. However, this study still has certain limitations, including the RNA seq sample size being relatively small and the reliance on RNAi. This study elucidated the tolerance mechanism of *A. pernyi* to beta-cypermethrin, providing a theoretical basis for subsequent research on the gene functions of *A. pernyi* through molecular approaches.

## Figures and Tables

**Table 1:** Toxicity bioassay of *A. pernyi* larvae against four insecticides.

Insecticides	Strains	Slope ± SE	LC_50_ (95% CL) (mg/L)	χ^2^ (*df*)	*p*
Beta-cypermethrin	Liaocanda9	2.76 ± 0.35	0.0080 (0.0070–0.0091)	10.21 (13)	0.68
	Kangda	2.96 ± 0.36	0.0047 (0.0038–0.0055)	13.30 (13)	0.42
Chlorantraniliprole	Liaocanda9	3.01 ± 0.35	12.12 (10.38–13.97)	14.89 (13)	0.31
	Kangda	3.42 ± 0.37	8.34 (7.19–9.41)	12.48 (13)	0.49
Imidacloprid	Liaocanda9	3.24 ± 0.37	0.52 (0.47–0.59)	4.80 (13)	0.98
	Kangda	3.84 ± 0.40	0.33 (0.28–0.36)	8.14 (13)	0.83
Thiamethoxam	Liaocanda9	2.63 ± 0.34	17.83 (14.85–20.58)	12.01 (13)	0.53
	Kangda	3.47 ± 0.42	12.08 (9.90–13.97)	9.52 (13)	0.73

**Table 2:** P450, CarE, and GST activities of Liaocanda9 and Kangda strains after treatment with LC_50_ beta-cypermethrin.

Group	P450 Activity (nmol/min/mg·prot)	GST Activity (μmol/min/mg·prot)	CarE Activity (μmol/min/mg·prot)
Liaocanda9	0.64 ± 0.01 c	210.13 ± 3.84 b	508.48 ± 9.50 b
Liaocanda9 + LC_50_	0.99 ± 0.03 a	288.80 ± 7.50 a	598.37 ± 9.86 a
Kangda	0.55 ± 0.01 d	177.33 ± 1.34 c	456.43 ± 6.16 c
Kangda + LC_50_	0.73 ± 0.01 b	201.65 ± 4.42 b	544.16 ± 9.16 b

Note: Different letters indicate significant differences in all groups based on ANOVA followed by Tukey’s HSD post hoc test (*p* < 0.05).

**Table 3:** SOD, POD, and CAT activities in Liaocanda9 and Kangda strains after treatment with LC_50_ beta-cypermethrin.

Group	SOD Activity (U/mL)	POD Activity (U/mL)	CAT Activity (U/mL)
Liaocanda9	275.99 ± 2.19 b	45.83 ± 0.45 b	7.82 ± 0.03 c
Liaocanda9 + LC_50_	293.55 ± 3.84 a	49.74 ± 0.14 a	9.80 ± 0.03 a
Kangda	239.62 ± 3.45 c	38.77 ± 0.33 d	7.50 ± 0.07 d
Kangda + LC_50_	267.17 ± 4.25 b	41.27 ± 0.15 c	8.80 ± 0.02 b

Note: Different letters indicate significant differences in all groups based on ANOVA followed by Tukey’s HSD post hoc test (*p* < 0.05).

## Data Availability

The data has been submitted to the NCBI database, with the access ID: PRJNA1440535. The original contributions presented in this study are included in the article/Supplementary Materials. Further inquiries can be directed toward the corresponding authors.

## Supplementary Materials

The following supporting information can be downloaded at: https://www.mdpi.com/article/10.3390/insects17040415/s1. Table S1. RT-qPCR primer information of differentially expressed genes. Table S2. Primer sequences for synthesis of dsRNA transcription templates. Table S3. Transcriptome data statistic.
