# Supplementary material for: Contributions of ABC Transporters and Cytochrome P450s to the Tolerance in Antheraea pernyi Against Beta-Cypermethrin
Source: Insects. 2026 Apr 13;17(4):415. doi: 10.3390/insects17040415 (PMC13116884; doi:10.3390/insects17040415)
Supplement: Supplementary file 1 [file insects-17-00415-s001.zip › insects-4217172-supplementary.pdf]

Contributions of ABC transporters and cytochrome P450s to the tolerance in *Antheraea pernyi* against beta-cypermethrin

Tianyi Zhang[1], Xin Chen[1], Junshan Chen[1], Peifeng Liu[1], Fengquan Liu[1], MiaoMiao Chen[1], Liang Xu[1], Shiwen Zhao[1], Xisheng Li[1,*]

[1]Sericultural Research Institute of Liaoning Province, Dandong 118100, China

[*]Corresponding Author

Table S1. RT-qPCR primer information of differentially expressed genes.

Gene	Direction	Primer Squence	Length
ABCA3	Forward	GTATCGGCAGCAGTATAGC	123
	Reverse	TGAACCACAGCACAGATG	
ABCG1	Forward	CACTGGTTTGGACATCGTAT	167
	Reverse	CGCCCTGATAACAACACAT	
ABCG5	Forward	CGAGTCTTCTGTTGTCTTGGA	191
	Reverse	GACGAACATCATAACCGCAATAG	
ABCG8	Forward	CCGTAATACTATCAGGCTTCT	132
	Reverse	CTTCAGGCAGAGTCCATC	
CYP9A22	Forward	CAATGGACACTGGATTCTCTAC	177
	Reverse	CGTCAGGATTCACAGCAAG	
CYP49A1	Forward	CGAAAGGGACACAGGTGATA	143
	Reverse	AAACGGTAAAGACGCAAAGG	
CYP313A4	Forward	GCATCTGGTCGTGTTCTAC	176
	Reverse	CGAGGTCCACTGCTAAATG	
CYP341B1	Forward	GAAGCATCCAGCGTCATT	175
	Reverse	CAGCGGTTTGTCTTTCTCA	
GSTD1	Forward	CATACTTGGAGATACCGTTCG	121
	Reverse	AAGCCATCATCATACAGCAC	
GSTT1	Forward	GTGGAAGGTGTTAGAACTGTTAC	131
	Reverse	GCTGATATGTCGGCAATAGTC	
CarEB1	Forward	GACACCATCCCAGCAGAAA	166
	Reverse	CCGCATCAGCACTACCAT	
CarEFE4	Forward	CGAACTCAGGTTTCAAGCA	79
	Reverse	ACACATCGGTCCATCCTC	
UGT	Forward	TTATCGCAGTAACGCCAAA	174
	Reverse	ATCAGAATGTCCAGGTGTAATC	
SOD	Forward	TCAACCCAGAACACAAAGA	111
	Reverse	CGACCATATCAATCCTGCT	
POD	Forward	CCGTATGAGTGACTGGTT	162
	Reverse	CCGCCGTATGTATTATTGC	
18S rRNA	Forward	GAACTACTGACATCACAGGCTAT	113
	Reverse	GCTCCTCCAGTCATAAGAACTAA	

Table S2. Primer sequences for synthesis of dsRNA transcription templates

Gene	Direction	Primer Squence	Length
dsABCG1	Forward	taatacgactcactatagggGCTTCAGCCCAGATGTCAGA	577
	Reverse	taatacgactcactatagggAGGATCGGTCCCATTCAAACA	
dsABCG5	Forward	taatacgactcactatagggAAGGATCCACTGATACTACTCCT	502
	Reverse	taatacgactcactatagggCCAGCATCCGCACATACAAC	
dsABCG8	Forward	taatacgactcactatagggCAATCGGCACTCGGACTGAC	504
	Reverse	taatacgactcactatagggTCGAAAATCAGGATCCGAACGA	
dsCYP9A22	Forward	taatacgactcactatagggTCTGGAGATGGTCAAATTGAAGT	504
	Reverse	taatacgactcactatagggTGTTTCGAATCCGGCGATGA	
dsCYP49A1	Forward	taatacgactcactatagggAGCCGTTCATGGAGATTCGT	511
	Reverse	taatacgactcactatagggTGCAACAGCAGCCACTCTC	
dsEGFP	Forward	taatacgactcactatagggGCGAGGGCGATGCCACCTAC	431
	Reverse	taatacgactcactatagggCACGCTGCCGTCCTCGATGT	

Table S3. Transcriptome data statistic

Sample	Raw reads	Raw bases	Clean reads	Clean bases	Error rate	Q20(%)	Q30(%)	GC(%)
D9CK1	43366580	6548353580	43094136	6471461883	0.0118	99.27	96.17	45.33
D9CK2	44243898	6680828598	43943534	6602891357	0.0118	99.28	96.3	45.15
D9CK3	42430136	6406950536	42144368	6328831904	0.0119	99.25	96.09	47.3
KDCK1	43739016	6604591416	43452118	6530647194	0.0119	99.26	96.08	46.82
KDCK2	47994876	7247226276	47693906	7166386457	0.0118	99.28	96.24	47.34
KDCK3	42353444	6395370044	42118854	6329847777	0.0117	99.31	96.34	46.98
D9T1	41971380	6337678380	41685470	6259620777	0.0119	99.24	96.07	48.46
D9T2	43798098	6613512798	43509682	6532821641	0.0118	99.27	96.2	46.51
D9T3	41722882	6300155182	41394998	6214508504	0.0118	99.25	96.11	48.42
KDT1	44812766	6766727666	44492600	6679322803	0.0118	99.28	96.26	46.27
KDT2	47073484	7108096084	46766846	7014465254	0.0117	99.3	96.38	45.9
KDT3	46120070	6964130570	45846510	6867570080	0.0118	99.29	96.29	46.36
